# REINFORCEMENT WITH THE LONG HEAD OF THE BICEPS TENDON IN LARGE AND MASSIVE REPAIRABLE ROTATOR CUFF TEARS: A PROSPECTIVE CASE SERIES

**DOI:** 10.1590/1413-785220253302e288796

**Published:** 2025-10-13

**Authors:** GUSTAVO DE MELLO RIBEIRO PINTO, CRISTIANO NABUCO DANTAS, MARCELO COSTA DE OLIVEIRA CAMPOS, JORGE HENRIQUE ASSUNÇÃO, MAURO EMILIO CONFORTO GRACITELLI, EDUARDO ANGELI MALAVOLTA

**Affiliations:** 1. Universidade Estadual do Rio de Janeiro, Hospital Universitario Pedro Ernesto (HUPE), Rio de Janeiro, RJ, Brazil.; 2. Universidade de Sao Paulo, Faculdade de Medicina Hospital das Clinicas (HCFMUSP), Sao Paulo, SP, Brazil.; 3. DASA, Hospital 9 de Julho, Sao Paulo, SP, Brazil.; 4. Hospital do Coração (Hcor), Sao Paulo, SP, Brazil.

**Keywords:** Rotator Cuff, Shoulder, Tendon Transfer, Autografts, Lesões do Manguito Rotador, Ombro, Transferência Tendinosa, Autoenxerto

## Abstract

**Introduction:**

The treatment of large and massive rotator cuff tears remains challenging. This study evaluates the efficacy and safety of reinforcement with the long head of the biceps tendon (LHBT) in patients with large and massive repairable rotator cuff tears.

**Methods:**

This is a prospective case series involving 25 patients who underwent open repair of large and massive rotator cuff tears with LHBT reinforcement. All patients were operated by the same surgeon and followed up for one year.

**Results:**

Pain scores (VAS: 6.6 ± 2.30 vs. 2.68 ± 2.73; p<0.001) and function scores (ASES: 36.86 ± 19.27 vs. 73.96 ± 23.73; p<0.001; UCLA: 13.04 ± 3.83 vs. 26.04 ± 7.39; p<0.001) improved significantly postoperatively compared to preoperatively. The biceps healing rate was 84%, while the rotator cuff retear rate was 60%. No complications related to the biceps or surgical site infections were documented.

**Conclusion:**

Reinforcement with the long head of the biceps tendon in the repair of large and massive rotator cuff tears shows satisfactory clinical outcomes and a low complication rate. Level of Evidence: IV, Case Series.

## RESUMO

**Introdução:**

O tratamento das lesões grandes e extensas do manguito rotador continua desafiador. Este estudo avalia a eficácia e segurança do reforço com o tendão da cabeça longa do bíceps (TCLB) em pacientes com lesões reparáveis grandes e extensas do manguito rotador.

**Métodos:**

Esta é uma série de casos prospectivos com 25 pacientes submetidos ao reparo aberto das lesões grandes e extensas do manguito rotador com reforço do TCLB. Todos os pacientes foram operados pelo mesmo cirurgião com seguimento de um ano.

**Resultados:**

Os escores de dor (EVA: 6,6 ± 2,30 vs. 2,68 ± 2,73; p<0,001) e função (ASES: 36,86 ± 19,27 vs. 73,96 ± 23,73; p<0,001; UCLA: 13,04 ± 3,83 vs. 26,04 ± 7,39; p<0,001) melhoraram significativamente no pós-operatório em comparação com o pré-operatório. A taxa de cicatrização do bíceps foi de 84%, enquanto a taxa de re-rotura do manguito foi de 60%. Não documentamos nenhuma complicação relacionada ao bíceps ou infecção no sítio cirúrgico.

**Conclusão:**

O reforço com o tendão da cabeça longa do bíceps no reparo de lesões grandes e extensas do manguito rotador apresenta resultados clínicos satisfatórios e baixa taxa de complicações. Nível de Evidência: IV, Série de Casos.

## INTRODUCTION

The treatment of large and massive rotator cuff tears remains challenging. In these injuries, after complete or partial tendon repair, the failure rates can reach up to 94% due to significant fatty degeneration, tendon retraction, reduced mobility, and muscle atrophy.^1,2^

Several procedures have been described to assist in the surgical repair of these lesions such as medialization of the footprint, anterior interval release, margin convergence and the use of synthetic or biological grafts in the repair.^3^ Superior capsule reconstruction (SCR), initially described for irreparable supraspinatus tears using autologous fascia lata graft, has been shown to reduce retear rates and improve tendon quality, as well as in SCR in repairable rotator cuff tears.^4^

An alternative to SCR is the use of the long head of the biceps tendon (LHBT) as an autologous graft. The transposition and incorporation of the biceps tendon into the rotator cuff repair adds native tissue rich in tenocytes and fibroblasts to the repair. Other advantages include reduced cost and decreased risk of disease transmission associated with allograft use; the lack of need for additional incisions preventing lower limb morbidity for fascia lata grafts; in addition to being technically more reproducible.^5^

The objective of this study is to evaluate the clinical and radiological outcomes of patients who underwent open repair of large and extensive rotator cuff tears with reinforcement using the long head of the biceps tendon.

## METHODS

This is a prospective case series in which all patients were operated on between January 14, 2022, and April 14, 2023, by the same surgeon with six years of experience in shoulder and elbow surgery and approved by the Institutional Ethics and Research Committee (5.154.677). All patients signed the consent form.

Patients met the following inclusion criteria on magnetic resonance imaging: large (≥ 3 cm) or massive (> 5 cm) posterosuperior rotator cuff tears according to the DeOrio and Cofield classification,^6^ intact LHBT, and fatty degeneration of the supraspinatus muscle ≤ 2 according to the Goutallier classification.^7^

Patients with a history of infection in the affected shoulder, inability to understand the preoperative questionnaires, patients who did not undergo at least one postoperative evaluation, tears where complete rotator cuff repair was not possible, and complete or partial tears > 25% of the LHBT diagnosed intraoperatively were excluded.

Clinical outcomes were assessed using the American Shoulder and Elbow Surgeons (ASES)^8^ and the University of California Los Angeles (UCLA)^9^ scores preoperatively and at 6 and 12 months postoperatively. The visual analog scale (VAS)^10^ for pain was applied at the same intervals, and additionally on the 1st and 14th postoperative days. The scores were administered by the principal investigator.

Radiographic evaluation included the Hamada^11^ classification and the measurement of the acromiohumeral distance (AHD) preoperatively and 6 months postoperatively, in an anteroposterior radiograph of the glenohumeral joint. The AHD was measured as the shortest distance from the inferior surface of the acromion to the superior face of the humerus.

Patients underwent preoperative and 6-month postoperative magnetic resonance imaging, evaluated by a musculoskeletal radiologist with 15 years of experience. Rotator cuff healing was classified according to Sugaya et al.^12^ categorized as healed (stages I/II/III) or not healed (stages IV/V). LHBT reinforcement healing was also categorized as healed or not healed.

### Surgical procedure

Patients were operated in a beach chair position using a surgical approach from the anterolateral border of the acromion, approximately 5 cm in length, followed by opening the interval between the anterior and middle portions of the deltoid with subsequent resection of the subdeltoid bursa. At this point, the integrity of the LHBT was assessed.

**Figure 1:**
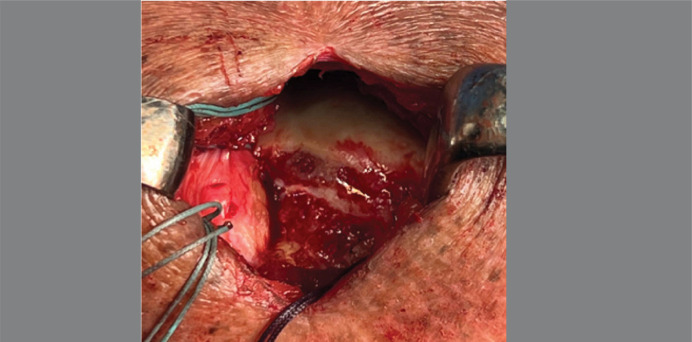
Cruentation and medialization of the greater tuberosity *footprint*.

Source: Author's personal fie.

Next, the pattern of the rotator cuff tear and the mobility of the tendons were analyzed to achieve a complete repair. For this, braided polyethylene #2 sutures were placed in the tendon. The greater tuberosity of the humerus was debrided, and the footprint was medialized for tendon reinsertion (Figure 1).

**Figure 2:**
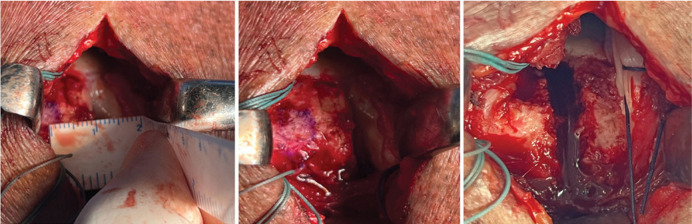
A) Measurement of the neo-bicipital groove distance; B) Marking of the neo-groove 1 to 1.5 cm posterior to the native groove (blue pen); C) Neo-bicipital groove with a depth of 3 mm.

Source: Author's personal fie.

**Figure 3:**
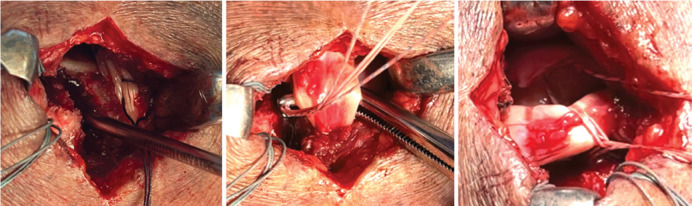
A) Placement of the metal anchor in the neo-groove; B) Passage of the sutures through the biceps; C) “Lasso loop”.

Source: Author's personal fie.

The proximal insertion of the LHBT was kept intact, and the transverse ligament was excised in the bicipital groove to allow posteriorization of the biceps tendon. The LHBT was repositioned and fixed with a Super Revo® FT 5.0 mm metal anchor with two Hi-Fi® #2 sutures (Conmed, Largo, FL, USA) in the supraspinatus footprint, in a neo-groove with a depth of 3 mm and located 1 to 1.5 cm posterior to the lateral border of the bicipital groove, created with the aid of a curette, while maintaining the arm at 10° flexion and 30° abduction (Figure 2A-C). One suture from the anchor was used for anchoring and repositioning the LHBT in the footprint with a “lasso loop” stitch described by Lafosse et al.^13^ and the other suture was tied around the tendon (Figure 3A-C).

**Figure 4:**
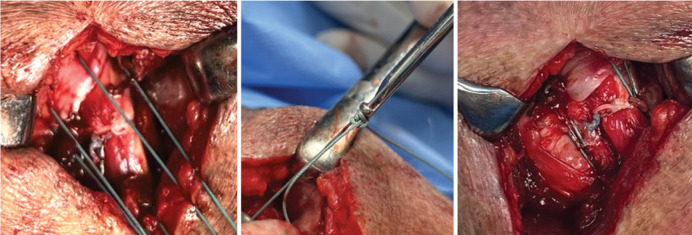
A) Tensioning of the rotator cuff over the tenotomized long head of the biceps in the neo-groove; B) “Nicky’s knot”; C) Final repair.

Source: Author's personal fie.

The rotator cuff repair was performed using the transosseous suture technique.^14,15^ The sutures were evenly distributed according to the size of the tear and tied with “Nicky’s knot” sliding knots.^16^ (Figure 4A-C).

### Postoperative follow-up

All patients used an abduction sling for 6 weeks but were encouraged to remove it during the day for personal care and to move the elbow, wrist, and fingers. After 6 weeks, patients were referred to physical therapy with guidance to perform assisted passive shoulder movement, and subsequently active movement. Sixteen weeks after surgery, patients began exercises to strengthen the rotator cuff and the scapular stabilizing muscles.

### Statistical analysis

Intrinsic patient variables, factors related to the injury, and the intervention were evaluated between the groups.

Continuous variables were assessed for normality using the Kolmogorov-Smirnov test and for homogeneity using Levene’s test.

Categorical data were presented in absolute and percentage values. Continuous data were presented as means and standard deviation and, when non-parametric also as median.

The results obtained by the assessment scales (UCLA, ASES, EVA and SF-12) at different follow-up times were compared by Analysis of Variance (ANOVA) and post-hoc analysis was performed with Bonferroni adjustment. The acromiohumeral distance preoperatively and at 6 months of follow-up were compared using the Wilcoxon test. The p value <0.05 was considered statistically significant. We used the SPSS version 20.0 program to analyze the data.

## RESULTS

In total, 46 patients were operated on, of which 25 patients underwent complete repair of large and extensive rotator cuff tears with TCLB reinforcement, 13 men and 12 women with a mean age of 61 ± 7.7 years, followed for a year (Table 1). Twenty-one patients were excluded intra-operatively for the following reasons: irreparability of the injury (7); LHBT injury > 25% (6); partial repair (2); and rotator cuff injury < 3 cm (6).

**Table 1:** Demographic Characteristics of the Patients.

Variables	n = 25
Sex, n(%)	
Male	13 (52)
Female	12 (48)
Age, years (mean ± SD)	61 ± 7.65
Affected side, n (%)	
Right	13 (52)
Left	12 (48)
Dominant side, n (%)	
Right	25 (100)
Left	0 (0)
Alcoholic, n (%)	13 (52)
Smoker, n (%)	2 (8)
Arterial hypertension, n (%)	13 (52)
Diabetes, n (%)	6 (24)
Hypothyroidism, n (%)	1 (4)
HIV, n (%)	3 (12)
Rheumatoid arthritis, n (%)	3 (12)

SD: standard deviation; n: number of patients.

The VAS scale decreased from 6.6 ± 2.3 to 2.7 ± 2.7 (p < 0.001) at 12 months. The ASES and UCLA scales evolved from 36.9 ± 19.3 and 13.0 ± 3.8 to 74.0 ± 23.7 and 26.0 ± 7.4, respectively (p < 0.001), at the end of the follow-up. Twenty-one (84%) patients surpassed the MCID in the 1-year postoperative evaluation using the ASES and UCLA scale.^16^ Four patients did not reach the MCID, of which only one patient was dissatisfied after a 12-month follow-up evaluation. (EVA: 7; ASES: 27; UCLA: 17) and was converted to reverse arthroplasty (Table 2).

**Table 2:** Preoperative and Postoperative Functional Outcomes.

	Mean	SD	p-value
**VAS**			p<0.001*
Preoperative	6.6	2.3	
1º day	5.2	2.1	
14º day	4.3	3	
6 months	3.2	2.8	
12 months	2.6	2.7	
**ASES**			p<0.001*
Preoperative	36.8	19.2	
6 months	66.7	24.2	
12 months	73.9	23.7	
**UCLA**			p<0.001*
Preoperative	13	3.8	
6 months	23.1	7.8	
12 months	26	7.3	
**SF-12 - Physical**			**p=0.001**
Preoperative	32.7	8.5	
6 months	39.5	11.3	
12 months	41.2	13.1	
**SF-12 - Mental**			**p=0.391**
Preoperative	45.8	13.7	
6 months	42.3	14.2	
12 months	43	14.4	

VAS: Visual analogue scale; ASES: American society of shoulder and elbow score; UCLA: University of California Los Angeles score; SF-12: Short Form Health Survey; SD: Standard deviation. *Post-hoc analysis: The VAS score in the preoperative period differs from that obtained on the 1st day, 14th day, 6 months, and 12 months (p<0.0001). The comparison of the periods preop x 14th day, preop x 6 months, preop x 12 months, 1st day x 6 months, and 1st day x 12 months showed p<0.0001; however, it was not significant for the periods preop x 1st day (p=0.1219), 1st day x 14th day (p=0.1486), 14th day x 6 months (p=0.1396), 14th day x 12 months (p=0.0306), and 6 x 12 months (p=0.1096). The UCLA score in the preoperative period differs from that obtained at 6 and 12 months (p<0.001). The comparison of the periods preop x 6 months and preop x 12 months showed p<0.0001; however, it was not significant for the period 6 x 12 months (p=0.005). The ASES score in the preoperative period differs from that obtained at 6 and 12 months (p<0.001). The comparison of the periods preop x 6 months and preop x 12 months showed p<0.0001; however, it was not significant for the period 6 x 12 months (p=0.016). The SF-12/physical score in the preoperative period differs from that obtained at 6 and 12 months (p<0.001). The comparison of the periods preop x 6 months and preop x 12 months showed p<0.001; however, it was not significant for the period 6 x 12 months (p=0.526).

The comparison of SF-12 quality of life scores preoperatively and postoperatively showed a statistically significant improvement in the physical aspect (p = 0.001) but not in the mental aspect (p = 0.391).

Patients were divided according to the Hamada classification, with a predominance of type I (68%) compared to type II (32%) (Table 3). The increase in acromiohumeral distance from preoperative to 6 months postoperative was from 6.28 mm to 6.92 mm, with no statistically significant difference (p = 0.202) (Table 4).

**Table 3:** Distribution of Patients by Radiological Classification.

Classification	n	%
Hamada		
I	17	68
II	8	32
Goutallier		
I	9	36
II	16	64

**Table 4:** Preoperative and postoperative acromiohumeral distance.

	Mean	SD	Median	p-value
Acromiohumeral distance (mm)				p=0.202
Preoperative	6.28	2.13	6.01	
6 months	6.92	2.73	6.66	

SD: Standard deviation.

All patients underwent magnetic resonance imaging at 6 months post-surgery to evaluate rotator cuff and long head of the biceps tendon healing. We observed a 60% retear rate of the rotator cuff (Sugaya IV = 6; Sugaya V = 9) and 84% of patients showed signs of LHBT healing.

No postoperative complications such as infection, anterior shoulder pain, crackling, fatigue, or anatomical deformity of the biceps due to retear were reported.

## DISCUSSION

The results of this prospective case series demonstrate that reinforcement of large and massive rotator cuff tears with the LHBT yields satisfactory outcomes with significant improvements in pain and UCLA and ASES scores.

The improvement in clinical scores is similar to that described by Rhee et al.^18^ who reinforced the repair of large and extensive rotator cuff tears with TCLB, finding an increase in ASES and UCLA scores between the preoperative and postoperative periods of 56.4 to 73.7 and 19.7 to 25.6, respectively. Cho et al.^19^ using the UCLA score, reported an increase in the score from 14.1 preoperatively to 32.6 points after surgery. In a similar way, but including lesions larger than 2 cm, Seo et al.^20^ reported that the ASES score between the pre- and postoperative periods improved from 43.2 to 91.9.

We assessed the impact of the results obtained with this technique on patients’ quality of life using the SF-12 questionnaire. We did not find similar studies in the literature that made this type of correlation. The physical component, which evaluates functional capacity, pain, and general health, showed significant improvement. However, the mental component, which relates to emotional and cognitive quality of life, did not show significant improvement. This component is influenced by sociodemographic aspects and patients’ mental health, which can be affected by pain and functional deficits, contributing to a low score.

Our study also observed significant pain improvement on the VAS. This finding is similar to that of Cheppalli et al.^21^ in a systematic review, which concluded that incorporating LHBT into the repair can improve VAS scores by up to 5 points.^21^

The rotator cuff retear rate in this study was 60%, which, although high, is considerably lower than that reported in the literature by other authors, ranging from 40 to 94%.^1,22^ Using a methodology similar to ours, Rhee et al. found comparable results, with a retear rate of 54.2%. When considering patients with fatty degeneration > 2 by the Goutallier classification, this retear rate increases to 75%.^18^ According to Malavolta et al.^23^ low fatty degeneration is an important prognostic criterion for achieving better clinical outcomes after rotator cuff repair; therefore, we chose to include only patients classified as Goutallier 1 (36%) and 2 (54%) of the supraspinatus.

We attribute the significant improvement in functional scores to the high healing rate of TCLB that was assessed on the MRI scan performed 6 months after surgery. We found an 84% healing rate of TCLB in the new groove, as did Veen et al. who reported in a systematic review, a biceps healing rate of 82% after 2 years of surgery.^24^ Restoration of the superior capsule in the treatment of large and extensive rotator cuff tears is essential to restore shoulder biomechanics, as demonstrated by Han el al.^25^ in which RCS with TCLB restores glenohumeral stability by re-centering the humeral head on the glenoid, even in irreparable injuries

Regarding the radiographic evaluation, the AHD remained stable without reducing the interval during follow-up (6.92 ± 2.7 mm). Kim et al. suggested that rotator cuff repair with LHBT reinforcement can exert a depressive force on the humeral head, however no study has shown a significant increase in AHD with this technique.^5,18^

Some complications related to LHBT surgical procedures, such as anterior shoulder pain, the appearance of Popeye’s sign, crackling, and fatigue have been described, although they rarely represent a functional problem for patients over time.^21^ Nevertheless, we did not document any of these complications or surgical site infections.

In our study, we reproduced the technique described by Kim et al.^5^ with adaptations for open rotator cuff repair. We opted to perform LHBT transposition with tenodesis in the new groove because we believe that keeping the LHBT intact after transposition allows the biceps muscle-tendon unit to theoretically add static force, keeping the humeral head centered and consequently ensuring good shoulder function.

We can cite some disadvantages of the study, such as the short follow-up time, which may not be sufficient to evaluate all possible complications and the long-term durability of the results. Another weakness is the need for a good-quality LHBT, which is often not seen in cases of massive rotator cuff tears. Thirdly, the small sample size may limit the generalizability of the results. Another important point is that only open rotator cuff repairs were performed, although the literature demonstrates that the results between arthroscopic and open techniques are similar.^26^ Finally, the absence of a control group prevents direct comparison with other repair techniques.

However, we used a methodology similar to other authors regarding functional assessment and repair healing.^18,27^ Moreover, Cañete San Pastor et al.^28^ believe that this technique would also have a therapeutic effect on patients with biceps pathology, thus eliminating the need for a good-quality LHBT.

The transposition and incorporation of the biceps tendon into the rotator cuff repair adds native tissue rich in tenocytes and fibroblasts for the repair. Other advantages include reduced procedure cost and disease transmission risk from allograft use, easy access to the LHBT during arthroscopy, and being technically more reproducible than other graft options for treating large and massive rotator cuff tears.^18^ This technique has proven to be safe and cost-effective, with the potential to increase rotator cuff repair healing rates and improve functional outcomes. However, future studies with larger samples and control groups are needed to confirm these findings.

## CONCLUSION

Reinforcement with the long head of the biceps tendon in the repair of large and massive rotator cuff tears offers satisfactory clinical outcomes. Patients showed significant improvements in pain scores, in the physical component of the Short Form Health Survey, and function after one year of follow-up, with a low complication rate.
